# Effect of general anesthesia on neonatal aEEG—A cohort study of patients with non-cardiac congenital anomalies

**DOI:** 10.1371/journal.pone.0183581

**Published:** 2017-08-31

**Authors:** Lisanne J. Stolwijk, Lauren C. Weeke, Linda S. de Vries, Maud Y. A. van Herwaarden, David C. van der Zee, Desiree B. M. van der Werff, Manon J. N. L. Benders, Mona Toet, Petra M. A. Lemmers

**Affiliations:** 1 Department of Neonatology, University Medical Center Utrecht, the Netherlands; 2 Brain Center Rudolf Magnus, University Medical Center Utrecht, the Netherlands; 3 Department of Pediatric Surgery, University Medical Center Utrecht, the Netherlands; 4 Department of Anesthesiology, University Medical Center Utrecht, The Netherlands; Massachusetts General Hospital, UNITED STATES

## Abstract

**Introduction:**

The aim of the current study was to determine the effect of general anesthesia on neonatal brain activity using amplitude-integrated EEG (aEEG).

**Methods:**

A prospective cohort study of neonates (January 2013-December 2015), who underwent major neonatal surgery for non-cardiac congenital anomalies. Anesthesia was administered at the discretion of the anesthetist. aEEG monitoring was started six hours preoperatively until 24 hours after surgery. Analysis of classes of aEEG background patterns, ranging from continuous normal voltage to flat trace in six classes, and quantitative EEG-measures, using spontaneous activity transients (SATs) and interSATintervals (ISI), was performed.

**Results:**

In total, 111 neonates were included (36 preterm/75 full-term), age at time of surgery was (median (range) 2 (0–32) days. During anesthesia depression of brain activity was seen, with background patterns ranging from flat trace to discontinuous normal voltage. In most patients brain activity was two background pattern classes lower during anesthesia. After cessation of anesthesia, recovery to preoperative brain activity occurred within 24 hours in 86% of the preterm and 96% of the term infants. Gestational age and the dose of sevoflurane were significantly associated with SAT-rate (F(2,68) = 9.288, p < 0.001) and ISI- durations during surgery (F(3,71) = 12.96, p < 0.001). Background pattern and quantitative EEG-values were not associated with brain lesions (χ2(4) = 2.086, ns).

**Conclusion:**

aEEG shows a variable reduction of brain activity in response to anesthesia in neonates with noncardiac congenital anomalies, with fast recovery after cessation of anesthesia. This reduction is related to gestational age and the dose of sevoflurane. The aEEG offers the opportunity to monitor the depth of anesthesia in the neonate.

## Introduction

EEG-derived monitors are most frequently used to investigate the anesthetic depth in children. However, EEG-derived monitors, such as the Bispectral Index Monitor, have only been investigated in a limited number of children and data is limited in infants[1–4]. Since EEG characteristics of infants are different from those of older children, a monitoring tool to guide anesthetic depth in neonates is not available[3,5–7]. A widely used monitoring tool for electrical brain activity in neonates admitted to the neonatology department is the amplitude-integrated EEG (aEEG). It offers continuous long-term monitoring of electrical brain activity, which is suitable to assess the background activity, detect sleep-wake cycling, and screen for seizures.

The cerebral activity is classified by background pattern recognition and voltage criteria[8]. Typically, the aEEG is discontinuous in preterm infants and gradually becomes continuous at term. The patterns burst suppression, continuous low voltage and flat trace have a poor prognosis in term infants[9,10]. Another prognostic indicator is sleep wake cycling[11], which is present from around 32 weeks of gestation to 44 weeks. This is recognized by sinusoidal variation in amplitude[8]. The presence of spontaneous activity transients (SAT) is a sign of brain immaturity and is observed in discontinuous and continuous brain activity. With increasing maturation the frequency of SATs decrease, although SATs are still observed in full-term neonates [12,13].

Neurodevelopmental outcome of patients with major non-cardiac congenital anomalies (NCCA), who require major neonatal surgery, warrants attention[14–16]. At two years of age, a cognitive and motor delay of up to 50% has been reported[17]. The causal pathway of this neurocognitive delay in children without a genetic syndrome is not completely understood. The clinical impact of inhalational anesthetics in infants and children is currently under investigation in three trials (PANDA, MASK and GAS-study)[18–20]. The first results show no differences in IQ-scores in later childhood, after a single and short exposure to anesthesia[18,19]. Nevertheless, this is only partly reassuring, since our patient cohort consists of young newborns undergoing major surgery, which might be of greater impact to the brain.

The aim of this study is to 1) evaluate the effect of general anesthesia on brain activity in preterm and term neonates using aEEG and 2) to review the effect of the anesthetic dose, brain injury and epileptic activity on the aEEG during anesthesia.

## Materials and methods

### Patients

In this prospective cohort study, patients with major non-cardiac congenital anomalies, requiring surgery in the neonatal period, were included between January 2013 to December 2015 at the Wilhelmina’s Children Hospital, University Medical Center Utrecht. This study was approved by the Medical Ethical Committee of the University Medical Center Utrecht (Utrecht, The Netherlands) for the use of clinically acquired data and the need for exclusively-written parental or guardian consent was waived. Inclusion criteria consisted of major non-cardiac congenital anomalies, surgery in the neonatal period, a postmenstrual age of 44 weeks or less during surgery. Exclusion criteria consisted of critical congenital heart disease and major congenital anomalies of the central nervous system.

### Amplitude-integrated EEG

**Fig 1:**
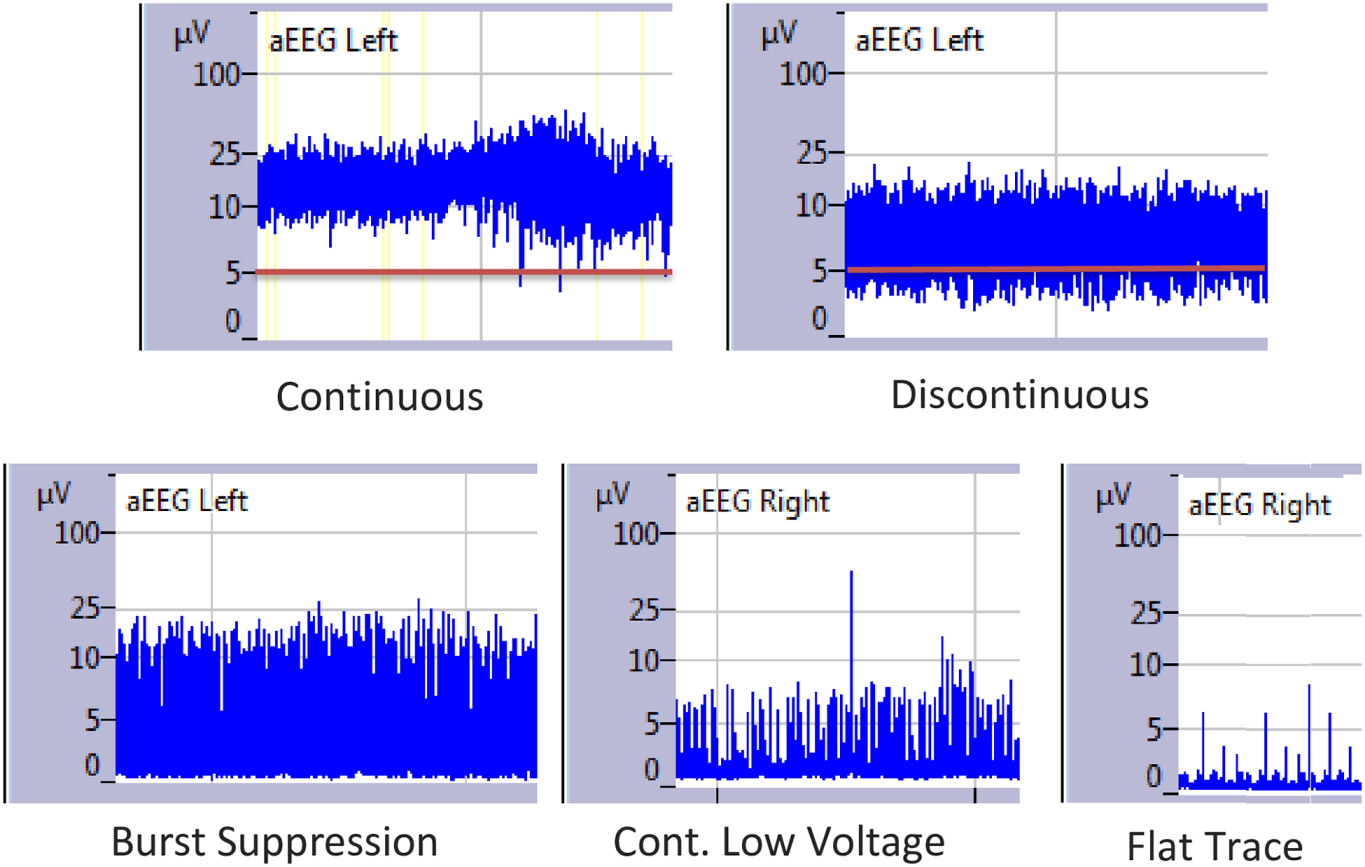
Examples of aEEG background patterns.

Patients were monitored with a two-channel EEG, using the BrainZ Monitor (BRM3, version, Natus CA, Seattle, USA). The BRM3 records a two-channel aEEG as well as a raw EEG from two electrodes over each hemisphere (F3-P3, F4-P4, according to the international 10–20 system of electrode placement). The amplitude ranges from 0 to 100 μV and is displayed on a semilogarithmic scale(Fig 1). Monitoring started six hours prior to surgery, continued during surgery and for 24 hours after surgery. Patients with a shorter duration of measurement were not excluded from analyses, when background assessment was feasible.

### Neuro-imaging

At hospital admission, cranial ultrasonography (cUS) was performed, in order to detect the presence of pre-surgical brain injury, and repeated postoperatively. A postoperative MRI was performed on a 3.0 Tesla whole-body Achieva system (Philips Medical Systems, Best, Netherlands) as part of routine clinical care. The scanning protocol included T1-, T2-, diffusion and susceptibility weighted imaging.

### Anesthesia

Anesthesia was administered at the discretion of the anesthetist. For the induction of anesthesia sevoflurane or isoflurane was used with an FiO_2_ of 40–100%. Atracurium besylate was used most often as a muscle relaxant, and sufentanil for pain medication.

### Data analysis

The aEEG background pattern and raw EEG signals were simultaneously assessed offline by three aEEG experts (M. Toet, L.S. de Vries and L.C. Weeke) using Analyze. Only recordings with an impedance <5 kΩ were analyzed and periods containing artifacts, such as nursing care, intubation at the operating room, and diathermy were excluded. The aEEG background patterns were classified according to Hellström-Westas et al.[9] as: continuous normal voltage (CNV), discontinuous normal voltage (DNV), burst suppression (BS), continuous low voltage (CLV), and flat trace (FT) (Fig 1). Epileptic activity was defined as evolving rhythmic activity for >10s on the raw EEG in the absence of artifacts and classified as single seizure, repetitive seizures, or status epilepticus[10]. Sleep-wake cycling (SWC) was classified as no SWC(no cyclic variation of the aEEG background), imminent SWC and normal SWC[11]. The time to return to baseline background activity and to SWC was documented up to 24 hours postoperatively.

For quantitative analyses the cross-cerebral EEG signal (P3-P4) was used. EEG-data were recorded at a sampling rate of 256 Hz. The recorded aEEG were assessed visually to identify marked artifacts, periods of high impedance, and other events (e.g. diathermy, blood sampling, care). Using in house developed software (Signalbase; version 7.8; University Medical Center Utrecht, Utrecht, The Netherlands) EEG data was analyzed. The following variables were calculated: number of spontaneous activity transients (SAT) per minute (SAT-rate) and the interval in seconds between SATs, the InterSatInterval (ISI).

**Fig 2:**
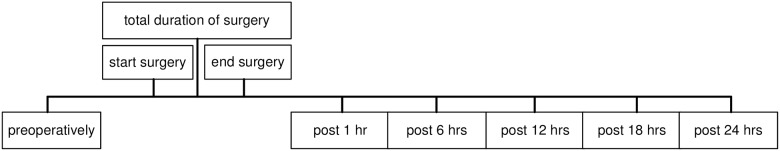
Epochs for analysis.

For analysis, nine epochs of 30 minutes were manually selected: preoperatively, first 30 minutes of surgery, last 30 minutes of surgery, total duration of surgery (variable duration), and 1hr, 6hrs, 12hrs, 18hrs and 24hrs after surgery (Fig 2). Start of surgery was defined as time of surgical incision (first 30 minutes) and end of surgery as time the surgeon finished (last 30 minutes of surgery). Other parameters, including heart rate (HR), arterial saturation, mean arterial blood pressure (MABP), perfusion index (PI), end-tidal carbon dioxide (etCO_2_), applied fraction of inspired oxygen (FiO_2_), respiration rate (RR), and end tidal sevoflurane (etSevo), were also simultaneously analyzed. These parameters were captured by in house developed software (Signalbase) at a sampling frequency of 1dp/sec.

Infants were classified preterm with a postmenstrual age < 37 weeks’ gestation at time of surgery. In children who underwent multiple surgical interventions, the first surgical intervention was included.

### Statistical analysis

Statistical procedures were performed using IBM SPSS statistics software package (IBM ^®^ SPSS ^®^ Statistics version 22, IBM Corp. Armonk, NY, USA). Data are presented as mean ± standard deviation (SD) or as median and range when indicated. A multivariable linear regression analysis was used to analyze the relationship during surgery between the SAT-rate or ISI, and postnatal age at surgery, birth weight z-score, dose of sevoflurane, sufentanil, propofol and duration of anesthesia. Correlations were checked using the Spearman correlation test. The Wilcoxon Signed Rank test was used to compare ISI- and SAT-values before, during and after surgery, a post hoc Bonferroni correction was applied to correct for multiple testing. A p-value < .05 was considered statistically significant.

## Results

### Study population

From January 2013 to December 2015, 114 infants with NCCA were admitted to the NICU for major neonatal surgery. Three infants were not eligible for inclusion in this study, since no perioperative aEEG was available due to logistic reasons. This resulted in a final sample of 111 infants being enrolled in this study (Table 1). Of these, 13 infants underwent multiple surgical interventions in the neonatal period.

**Table 1:** Demographic and surgical details of included patients.

	n = 111
**Gender (male, %)**	59 (53%)
**Preterm (n, %)**	36 (32%)
**Gestational age (weeks)**	38.28 (28–42)
**Birth weight z-score**	-0.50 (-3.12–2.00)
**Apgar score**	
At 1 minute	9 (2–10)
At 5 minutes	10 (2–10)
At 10 minutes	10 (6–10)
**Congenital abnormality, n(%)**	
Esophageal atresia	28 (25%)
Gastroschisis / omphalocele	16 (15%)
Intestinal atresia / volvulus	34 (31%)
Anorectal malformation	11 (10%)
Urogenital malformation	8 (7%)
Other	14 (12%)
**Surgery**	
**Postnatal age (days)**	2 (0–32)
**Duration anesthesia (minutes)**	186 (60–563)
**Duration surgery (minutes)**	119 (15–475)
**Multiple surgical interventions (patients)**	13(12%)
**Type of surgery**	
Laparotomy n(%)	49 (44%)
Laparoscopy n(%)	33 (30%)
Thoracoscopy n(%)	29 (26%)
Laparoscopy and thoracoscopy n(%)	1 (1.6)
**Arterial line n(%)**	95(86%)

Data are displayed in median[range], unless otherwise indicated

### aEEG background patterns

The median duration of aEEG recorded preoperatively was 5 hours and 24 minutes (range 3 minutes to 24 hours), seven patients had no preoperative measurement due to emergency surgery (n = 5) and logistic (n = 2) reasons. In seven patients sleep wake cycling could not be determined, because the duration of the preoperative measurement was too short (3 to 60 minutes).

**Fig 3:**
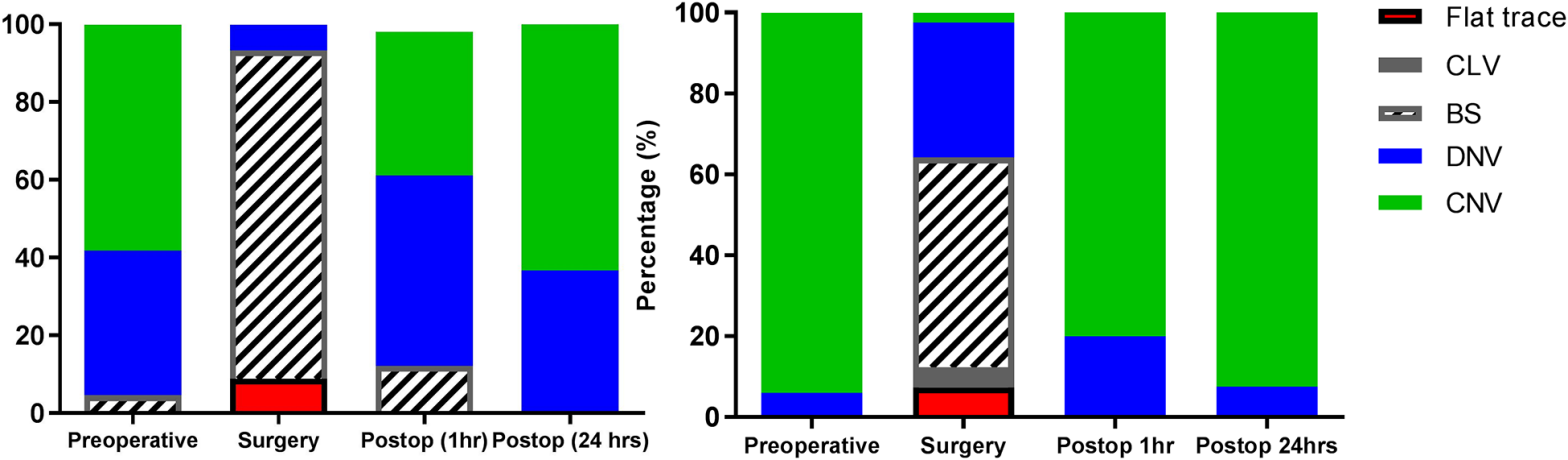
Predominant background pattern before, during and after surgery.

Pre-operatively, preterm infants (GA 34.7[28.1–36.9] weeks) had a continuous normal voltage background pattern in 58%, discontinuous normal voltage 37% and burst suppression 5%. During entire duration of surgery brain activity was depressed in all preterm infants. Postoperatively, 86% of the preterm infants returned to their preoperative background pattern within 24 hours, of which 60% within one hour. Sleep-wake cycling returned to preoperative patterns in 69%, and stayed imminent in 11% (Fig 3). Excluding patients who received midazolam postoperatively (n = 17)(Table 2), 87% returned to their preoperative background pattern within 24 hours, of which 65% within one hour. Sleep-wake cycling returned to preoperative patterns in 78%.

**Table 2:** Detailed characteristics of medication administered before, during and after surgery.

n = 129	Patients n(%)	Absolute dosage	Dosage/kg	Dosage/kg/hr
**Preoperative**				
Midazolam	9(7%)			0.05[0.04–0.1]
Morphine	29(22%)		0.37[0.23–1.00]	
**Surgery**				
*Anesthetic*				
Sevoflurane n(%)	126 (98%)	1.26[0.04–2.5]	NA	NA
Isoflurane n(%)	3(2%)	0.5[0.4–0.6]	NA	NA
Propofol, mg/kg n(%)	24 (19%)	10[2–20]	3.26[0.8–10.17]	1.06[0.23–3.41]
Midazolam n(%)	5(4%)	1.5[0.5–2.75]	0.53[0.15–0.88]	
*Pain medication*				
None	3(2%)			
Sufentanil	124 (96%)	2.25[0.25–12.50]	0.84[0.09–4.87]	2.47[0–32.45]
Bupivacaine*	31 (24%)	1.75[0.71–3.65]	0.56[0.19–2.37]	0.19[0.06–0.8]
Morphine	51 (40%)**	0.13[0.04–2.75]	0.06[0.02–1.17]	0.19[0.01–0.49]
*Muscle relaxant*				
None	6(5%)	-	-	-
Atracurium	106(82%)	4.0[1–15.50]	1.39[0.33–4.68]	3.43[0.54–41.91]
Rocuronium	16(12%)	4.5[1–10]	1.63[0.48–3.68]	4.34[1.73–17.05]
Suxamethonium	1(1%)	5.0	1.24	3.69
**Postoperative**				
Midazolam	38(29%)	-	-	0.05[0.03–0.28]
Morphine	101(78%)	-	0.28[0.22–0.62]	-
Bupivacaine	29(22%)			

Postoperative period was defined as 24 hours after end of surgery.

Sevoflurane in %, propofol mg/kg, midazolam mg/kg, sufentanil ug/kg, morphine mg/kg.

*Bupivacaine was administered through epidural at a continuous drip of 0.33mg/kg

**Fig 4:**
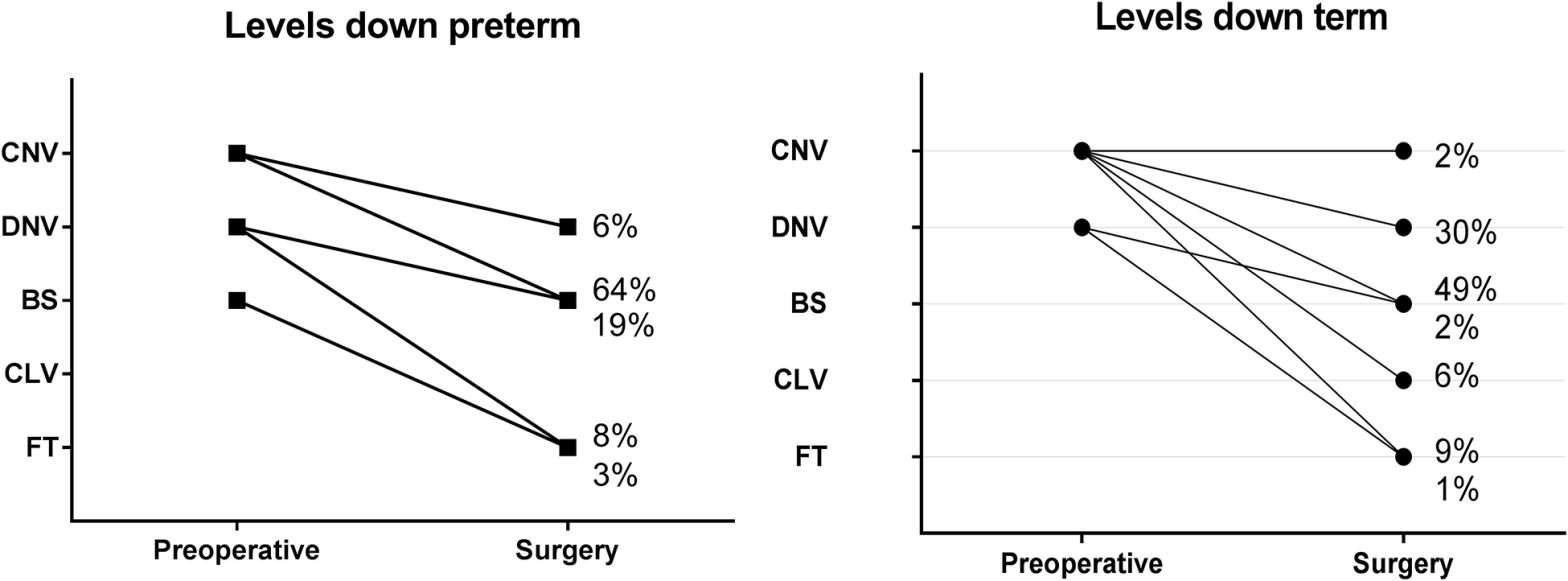
Degree of background pattern depression during surgery.

Of the term infants (39.1[37.0–41.9 weeks], 94% had a continuous normal voltage and 6% discontinuous normal voltage prior to surgery(Fig 3). In most patients the background pattern regressed two classes in comparison to preoperatively (preterm 67%, term 49%)(Fig 4). Of the two term patients with a predominant flat trace, one was diagnosed with a syndrome and the other had received a high dose of propofol (15 mg absolute dosage; 4.3 mg/kg). Postoperatively, 98% recovered to continuous normal voltage within 24 hours, of which 78% within one hour (Fig 3). Sleep-wake cycling returned to a normal pattern in 57%, and was imminent in 40%. Postoperatively, no difference in the administered dose of pain medication for the different background patterns and return of sleep-wake cycling was found (Kruskal Wallis, (H(1) = 2.220, p = 0.136)).

Excluding patients who received midazolam postoperatively (n = 22), 95% returned to continuous normal voltage within 24 hours, of which 84% within one hour. Sleep-wake cycling returned to a normal pattern in 95%.

During anesthesia nine term infants had a severe reduction in brain activity, from continuous normal voltage to continuous low voltage or flat trace. Of these, six patients received propofol during surgery (67% versus 19% all infants). No other common explanatory factors were found.

### Epileptic activity

Preoperatively, none of the patients had known or suspected seizure activity. In 11 patients epileptic activity was identified. In four infants epileptic activity (2 single seizures, 2 repetitive seizures) occurred during surgery, of which one directly after administration of sevoflurane during the induction of anesthesia (end tidal concentration sevoflurane 2.5–5%). Eight infants had seizures postoperatively (6 single seizure, 2 repetitive seizures). One infant had clinical seizures, and was diagnosed with a thalamic infarction, the other seven had subclinical seizures. No correlation with background pattern or brain injury was found. Four patients were suspected to have a genetic syndrome, one was diagnosed to have Moebius syndrome.

### Quantitative EEG analysis

**Fig 5:**
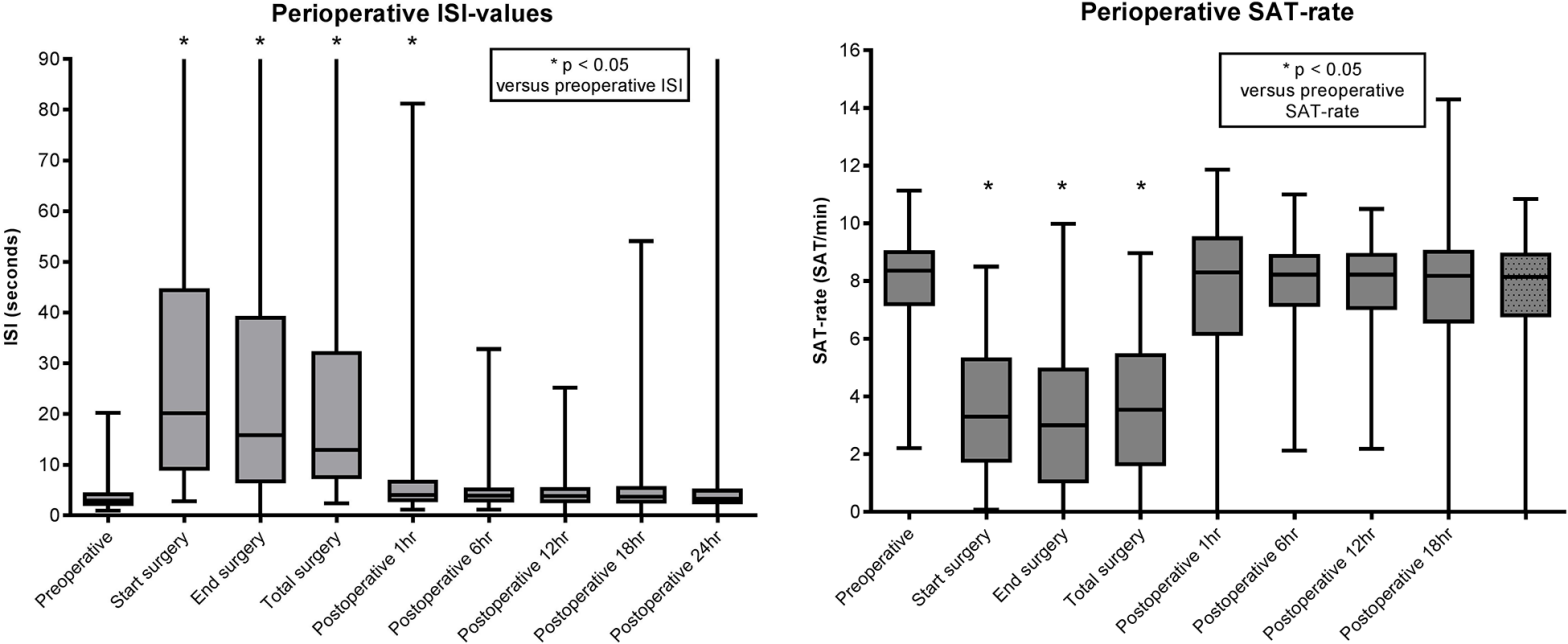
Median ISI-values and SAT-rates of all patients.

In preterm infants, ISI-durations were significantly longer during surgery (median ISI during total surgery period 33 seconds[3–571]) versus preoperative ISI (4 seconds[1–113]), Wilcoxon’s Signed rank test T = 29.0, r = -0.810, p<0.001 (Fig 5). One hour after surgery ISI were not significantly longer (median ISI 1hr: 5 seconds[1–75]) in comparison to preoperative ISI, T = 553, r = -0.304, p = 0.055 (Fig 5).

SAT-rates were significantly reduced during surgery (median SAT-rate during total surgery period: 2.23/minute [0.08–8.50]) than preoperative SAT-rates (7.94/minute[0.29–10.36]), T = 773, r = -0.850, p < 0.001 (Fig 5).

In term infants, ISI-durations were significantly longer during surgery (median ISI during total surgery period 14 seconds[3–253]) versus preoperative ISI (2 seconds[1–16]), Wilcoxon’s Signed rank test T = 2.0, r = -0.868, p<0.001 (Fig 5). One hour after surgery ISI-durations were significantly longer (median ISI 1hr: 3 seconds[1–81]) in comparison to preoperative ISI, T = 1731, r = -0.578, p < 0.001 (Fig 5).

SAT-rates were significantly reduced during surgery (median SAT-rate during total surgery period: 4.30/minute [0.16–8.50]) than preoperative SAT-rates (8.45/minute[2.67–11.13]), T = 2049, r = -0.844, p < 0.001 (Fig 5).

### ISI and background patterns

ISI-values during surgery correlated with the background pattern classification, showing that ISI-values of a continuous normal voltage pattern (median ISI 2.77 seconds[2–4]) were significantly shorter than a discontinuous normal voltage pattern (ISI 4.72 seconds[3–7]), and these were significantly shorter than a burst suppression (ISI 21.21 seconds[11–85]). Values during burst suppression were significantly lower than flat trace (ISI 76.95 seconds[25–215])(S1 Fig).

A factor significantly influencing the ISI-values was the type of procedure: ISI-values were significantly longer during a thoracoscopic procedure (median 28.4 seconds [3–571]) than during non-thoracoscopic surgery (median 15.3 seconds [3–393]), U 1121.00, p < .005, r = -0.26, z = -2.910 (S1 Table).

### Multivariable linear regression analysis SAT-rate and ISI during surgery

In the multivariable analysis, correcting for gestational age, the dose of sevoflurane showed a significant linear relation with SAT-rate and ISI during surgery. In particular, sevoflurane was positively related to ISI (β = 0.531, F(3,71) = 12.96, p < 0.001, R^2^ = 0.364, R^2^_adjusted_ = 0.336) and negatively associated with SAT-rate (β = -0.420, F(2,68) = 9.288, p < 0.001, R^2^ = 0.291, R^2^_adjusted_ = 0.259). Birth weight z-score, postnatal age at time of surgery, duration of anesthesia, the dose of sufentanil and the administration of propofol were not significantly associated to quantitative EEG measures.

### aEEG and brain injury

Preoperatively, 11 patients had brain injury on their ultrasound scan: one periventricular hemorrhagic infarction, one cerebellar lesion and nine infants inhomogeneous echogenicity, suggestive of punctate white matter lesions. Of all infants, in 58% parenchymal lesions were present on MRI and in 37% non-parenchymal injury. MRI-abnormalities were not significantly associated with the different background patterns or ISI and SAT-rate during surgery (Fisher-Freeman-Halton Exact Test: χ2(4) = 2.086, ns). For all data, see S2 Table.

## Discussion

Our study investigated the effects of general anesthesia on brain activity measured by aEEG in a neonatal cohort. The main findings were that the aEEG showed a transient, but very variable reduction of brain activity in neonates with major non-cardiac congenital anomalies. In most patients the background patterns decreased two classes during anesthesia. This depression in brain activity ranged from a flat trace to a discontinuous normal voltage. After cessation of anesthesia, 60% of the preterm and 78% of the term infants recovered within one hour after surgery to their preoperative background pattern. Within 24 hours, the background pattern of 86% of the preterm and 96% of the term infants had recovered. The gestational age and the dose of sevoflurane were significantly associated with the level of reduction in brain activity. Background patterns and quantitative EEG-measures during surgery were not associated with brain lesions and the occurrence of seizures.

There are no previous studies assessing the effect of anesthesia on the aEEG in a cohort of neonates during surgery[21]. Reports on EEG-derived measurements during anesthesia are rare in children, and limited in neonates [2,3,22–25]. In search of a neonatal device, diverse anesthetic depth monitors contain an algorithm based on adult EEG data. Since EEG parameters in neonates differ greatly from older children and adults, these devices cannot be used [3,26,27]. Therefore, we decided to investigate the use of the aEEG. The aEEG is commonly used in neonatal practice, and extensive knowledge has been gained on the use of aEEG in neonates. Although quantitative data rather than patterns may be preferred, the described background patterns can assist the pediatrician and anesthesiologist to intervene immediately.

In our quantitative analysis the Spontaneous Activity Transients were used. This endogenous activity is important for brain development[13]. These SAT’s are decreasing with GA, in number and amplitude approaching term equivalent age and remain detectable at least until week 44[28]. In our study the SAT-rate was able to make a distinction between the different background patterns and was therefore considered suitable for further analysis.

The reduction of brain activity, expressed by an increased interSATinterval and a decreased SAT-rate, was inversely related to the dose of sevoflurane. This is as expected, since this anesthetic causes a dose-dependent cortical inhibition by GABA stimulation[29]. It is of interest to observe such a large variation in brain activity, given the dose range used in our hospital. One of the guidelines for the appropriate dosage of sevoflurane is the Minimal Alveolar Concentration (MAC), which is 3.3% in the neonatal age group[30]. The range of the dose of sevoflurane used in our study was substantially lower: 0.4 to 2.5%. In a study performed by McKeever et al. no changes in aEEG were observed in children between one month and two years of age with a dose between 0.75MAC and 1.25MAC[22], which is in contrast to our results. A possible explanation could be the younger age of our cohort. Previous analyses during anesthesia has been performed by using EEG in infants[5] or only comprising a few neonates [2]. Furthermore, the reduction was age-dependent, even observable in the small gestational age width of our patient population[5].

Early brain activity is important for neuronal development[31]. Anesthesia causes a relatively short depression of brain activity with a rapid recovery postoperatively. Still, Backeljauw et al. found a decreased language comprehension and performance IQ in children exposed to anesthetics before the age of four[32]. In our cohort, severe brain lesions were not related to perioperative background patterns, SAT-rate or ISI-values.

Epileptic activity was identified on EEG in 11 neonates. One infant had been diagnosed with clinical seizures during admission. These seizures were not more prevalent in infants with a more depressed background pattern or with a higher prevalence of brain lesions. One child had a single seizure directly after the start of sevoflurane. This epileptiform activity has been described previously, and seems to be related to the speed of induction. Rapid induction is associated with a higher incidence of epileptiform discharges in comparison to a gradual induction[33,34]. Worldwide, concerns have been raised on the potential harmful effects of general anesthesia on the young infant’s brain. Awaiting the results of the clinical trials The Food and Drug Administration and the American Academy of Pediatrics recommended to reduce the overall drug dosage of anesthetics in young children. However, little is known about the immediate and long-term effects of clinical levels of volatile anesthetics on the developing brain. In general, the dose of anesthesia is based on the ‘clinical judgment of the anesthetist’[7,35]. Monitoring neonatal brain activity during anesthesia could ensure adequate dosing. Anesthetic depth monitors have been shown in adults to reduce the amount of anesthetic drugs used, reduce awareness and shorten recovery[3].

To be able to determine the adequate depth of anesthesia, we have to decide what level of depression in brain activity is sufficient. Do we agree that a discontinuous normal voltage is adequate anesthesia, in case of a continuous pattern preoperatively? Or, do we prefer to avoid any stress in the infant and lower the brain activity to a flat trace? One of the definitions of anesthesia is a loss of consciousness, amnesia, immobility and a reduction in the reflex autonomic responses associated with noxious stimuli[6]. The EEG measures cortical brain activity, which gives an indication of consciousness. This is of important additional value to other clinical parameters, such as HR, RR and MABP. Since for example, a movement response to a noxious stimulus is mediated by the subcortical brain(37).

One of the limitations of our study is the lack of a standardized anesthesia protocol. Anesthesia was administered at the discretion of the anesthesiologist, which reflects normal practice. The strength of this study is that the aEEG is commonly used in neonatal practice and our results add to the literature data on describing the clinically used background pattern. The aEEG offers continuous, bedside monitoring and the data can readily be interpreted by pediatricians and anesthesiologists. The study comprises a cohort with a small gestational age width, which is important since we know that cerebral maturation influences the EEG and the neonatal brain is developing fast.

The future perspective is to correlate the background pattern during anesthesia to long-term neurodevelopment. The time to recover to a normal background pattern and the onset of sleep-wake cycling has been proven in patients with hypoxic-ischemic encephalopathy to be important prognostic factors of outcome. Since the depressed pattern during anesthesia is iatrogenic and recovery after cessation is rapid, the prognostic value may limited. Surgery cannot easily be postponed or avoided, therefore studies on early childhood anesthesia for surgery and the effect on long-term cognitive function are needed.

In conclusion, general anesthesia in neonates causes a variable reduction in brain activity. This reduction is transient and recovery to the preoperative level of brain activity occurs rapidly. The depressant effect of sevoflurane is proportional to the dose, even observed in the small dose range in our study population. The aEEG offers the opportunity to monitor the depth of anesthesia in the neonate. The question remains which depth based on aEEG we aim for during anesthesia.

## Supporting information

S1 Table Differences between thoracoscopic and non-thoracoscopic procedures. (DOCX)

S2 Table Supporting information. (XLSX)

S1 Fig Correlation ISI-values and background patterns. (DOCX)
