# Supplementary material for: Effect of general anesthesia on neonatal aEEG—A cohort study of patients with non-cardiac congenital anomalies
Source: PLoS One. 2017 Aug 31;12(8):e0183581. doi: 10.1371/journal.pone.0183581 (PMC5578644; doi:10.1371/journal.pone.0183581)
Supplement: S1 Table — (DOCX) [file pone.0183581.s001.docx]

**Supplemental Table 1. Differences between thoracoscopic and non-thoracoscopic procedures**

|  | **Thoracoscopy** | **No thoracoscopy** | **p^#^** |
| --- | --- | --- | --- |
| **Gestational age (mean±SD)** | 37.48±2.66 | 37.32±2.82 | ns |
| **Postnatal age** | 3[0-30] | 2[0-30] | ns |
| **Birth weight z-score** | -0.36[-3.12-2.00] | -1.1[-2.10-1.74] | 0.056 |
| **Duration of anesthesia** | 227[78-490] | 160[60-563] | <.001 |
| **Propofol (%)** | 18% | 20% | ns* |
| **Arterial CO_2_ (mmHg)** | 50[34-84] | 42[30-80] | <.001 |
| **Sevoflurane** | 1.50%[0.4-3.0] | 1.50%[0.3-2.9] | ns |
| **Sufentanil (mg/kg/hr)** | 4.78[0.5-30] | 1.44[0-32] | <.001 |

Data in median[range], unless otherwise indicated. ^#^Mann Whitney *U* test with post-hoc Bonferroni, *Chi-square test.
