# Supplementary material for: Effect of general anesthesia on neonatal aEEG—A cohort study of patients with non-cardiac congenital anomalies
Source: PLoS One. 2017 Aug 31;12(8):e0183581. doi: 10.1371/journal.pone.0183581 (PMC5578644; doi:10.1371/journal.pone.0183581)

Supplemental figure 1. The correlations of the IBI-durations during surgery with sevoflurane and gestational age and the correlation SAT-values during surgery with sevoflurane and gestational age.


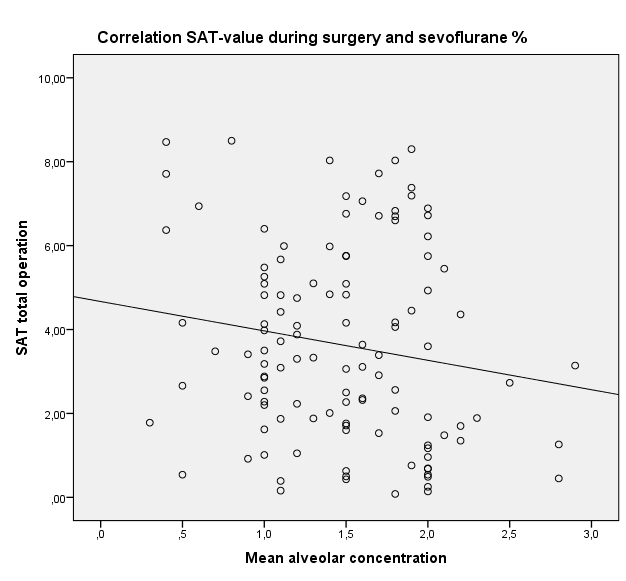


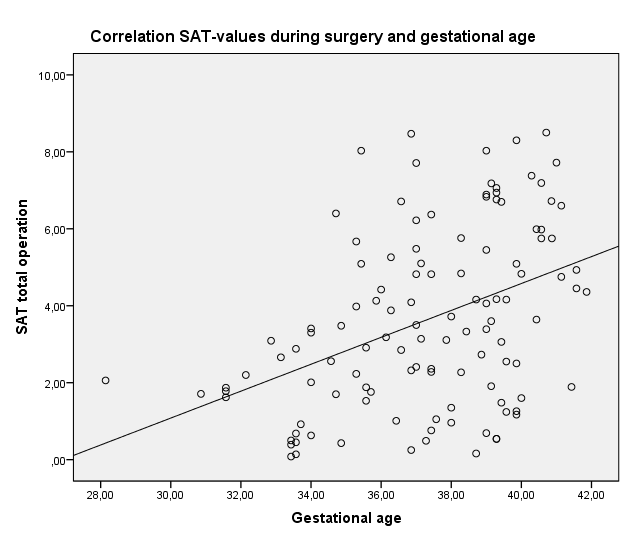


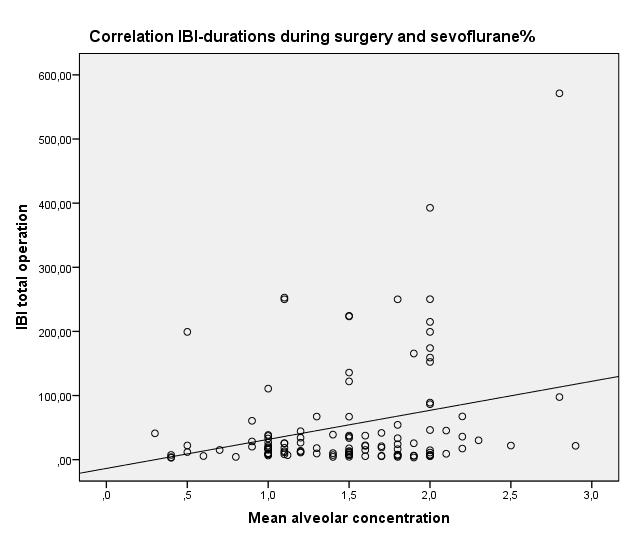


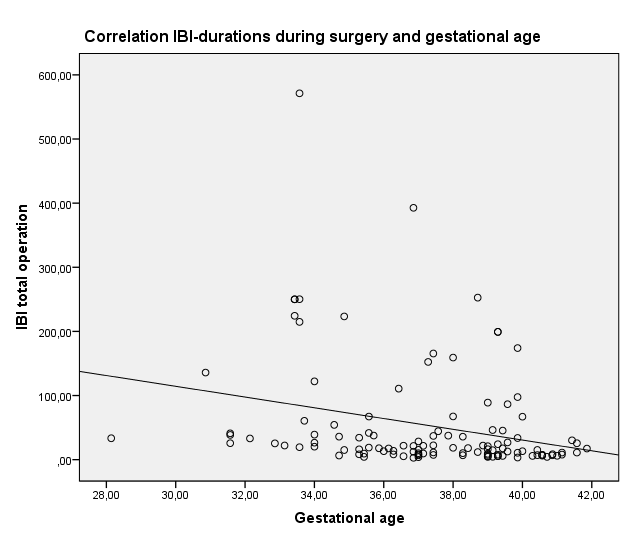

Supplement: S1 Fig — (DOCX) [file pone.0183581.s003.docx]
